# Enhanced Filtration Efficiency of Natural Materials with the Addition of Electrospun Poly(vinylidene fluoride-co-hexafluoropropylene) Fibres

**DOI:** 10.3390/ma16062314

**Published:** 2023-03-14

**Authors:** Gregor Filipič, Luka Pirker, Anja Pogačnik Krajnc, Marjan Ješelnik, Maja Remškar

**Affiliations:** 1 Jozef Stefan Institute, Jamova Cesta 39, 1000 Ljubljana, Slovenia; 2 Department of Electrochemical Materials, J. Heyrovsky Institute of Physical Chemistry, Dolejškova 3, 182 23 Prague, Czech Republic; 3 Faculty of Mathematics and Physics, University of Ljubljana, Jadranska Ulica 19, 1000 Ljubljana, Slovenia; 4 smartMelamine d.o.o., Tomšičeva Cesta 9, 1330 Kočevje, Slovenia

**Keywords:** filtration efficiency, electrospun fibres, aerosols, masks, PVDF

## Abstract

Pollutants and infectious diseases can spread through air with airborne droplets and aerosols. A respiratory mask can decrease the amount of pollutants we inhale and it can protect us from airborne diseases. With the onset of the COVID-19 pandemic, masks became an everyday item used by a lot of people around the world. As most of them are for a single use, the amount of non-recyclable waste increased dramatically. The plastic from which the masks are made pollutes the environment with various chemicals and microplastic. Here, we investigated the time- and size-dependent filtration efficiency (FE) of aerosols in the range of 25.9 to 685.4 nm of five different natural materials whose FE was enhanced using electrospun poly(vinylidene fluoride-co-hexafluoropropylene) (PVDF) fibres. A scanning electron microscope (SEM) was used to determine the morphology and structure of the natural materials as well as the thickness of the PVDF fibres, while the phase of the electrospun fibres was determined by Raman spectroscopy. A thin layer of the electrospun PVDF fibres with the same grammage was sandwiched between two sheets of natural materials, and their FE increased up to 80%. By varying the grammature of the electrospun polymer, we tuned the FE of cotton from 82.6 to 99.9%. Thus, through the optimization of the grammage of the electrospun polymer, the amount of plastic used in the process can be minimized, while achieving sufficiently high FE.

## 1. Introduction

Air pollutants such as heavy metals [1,2], nanoparticles [3], PM_2.5_, and PM_10_ [4,5] are suspended and transported through air as aerosols. The inhalation of such aerosols can lead not only to the development of respiratory diseases and lung cancer but also to a higher risk of cardiovascular and pulmonary diseases, reproductive and developmental problems, and a shorter lifespan in general [1,4,6,7,8,9]. Airborne droplets and aerosols can also carry other types of threats apart from inorganic pollutants, such as viruses and bacteria, and, thus, can spread highly infectious diseases. A recent example is the spread of the SARS-CoV-2 virus, which caused the COVID-19 pandemic in the beginning of 2020. To protect oneself against the airborne infections and pollutants, respiratory protection can be used. The spread of the SARS-CoV-2 virus was mitigated with the use of various personal protection equipment (PPE), such as masks, facepiece respirators, and barriers, to reduce and even stop the transmission of aerosols and droplets between individuals [10,11,12]. The need for PPE yielded a high mask production, and improvised solutions of different levels of protection were proposed [11,13,14].

With the increased production and consumption of PPE, the amount of non-recyclable waste has increased as well [15,16]. Face masks typically contain materials such as polypropylene, polyethylene, and polyurethane, which pose a hazard for the environment due to their corrosion resistance, chemical stability, and inability to be degraded by micro-organisms [17]. Some PPE is not discarded in the appropriate land fields, but is rather discarded into seas, rivers, and other parts of the environment, where it pollutes the environment with various chemicals and microplastics [18]. To mitigate the effect of the discarded PPE on the environment, alternative materials have been discussed and proposed, which would have similar properties as certified PPE, i.e., filtration efficiency, breathability, and comfortability [19]. Natural and bio-degradable materials would certainly remove the negative environmental influence.

One possibility is to use natural materials, such as cotton or linen. Differently woven cotton has already been tested in a previous study, but its filtration efficiency (FE) is lower compared to certified PPE [11,20]. For natural fabrics to replace current PPE, they need to stand up to current PPE standards. A possible solution to increase the FE of such material is to use a minimal amount of electrospun polymer fibres as an additional filtration layer [21,22,23].

With the proper selection of the polymer for electrospinning, one can ensure that the fibres have a static charge that creates a non-uniform electrical field [24,25,26]. Due to the electrical field, a force is imposed on the particles that have a net charge or an induced dipole. These forces then retard the progress of the particles by increasing the probability of an impact with a fibre due to diffusion and/or direct contact with the fibre’s surface. A theoretical model predicts that these kinds of electret fibres can successfully capture different viruses across a broad range of species and environments [27]. Without the electric charge, the fibres would have a substantial lower impact on the overall FE [12]. The lack of a static charge in natural fabrics is also one of the drawbacks when considering them for filtration applications. As polyvinylidene fluoride (PVDF) polymer charges up during electrospinning, we chose its fibres as anintermediate filter layer.

Electrospun PVDF polymer fibres possess desirable attributes such as chemical stability, mechanical strength, and biocompatibility [28,29], which are necessary due to the continuous mechanical deformations and exposure to humid air during breathing that face masks undergo. PVDF’s high hydrophobicity provides an additional mechanical barrier to prevent aerosol penetration and potential infection of the wearer. The electrospun PVDF fibres possess an intrinsic and long-lasting surface charge that results in increased filtration efficiency through electrostatic attraction. Additionally, the fibres’ small diameter, typically less than 200 nm, enhances the slip effects around them. Furthermore, the fibres exhibit superior electric charge retention compared to meltblown PP [30]. While the electrostatic charge can decay in humid conditions, a self-electrostatic-charging mechanism through triboelectrification has been reported, extending the lifespan of the electrospun filter layer for up to 60 h [31,32]. Due to their chemical and thermal resistance, the reusability potential of the designed face mask can be further investigated. The electrospun PVDF fibres can be electrostatically recharged (e.g., corona discharge) after the appropriate disinfection methods, restoring the mask’s filtration efficiency [25,33,34,35].

In the present study, we aimed to reduce the environmental impact of respiratory PPE by testing the FE of different textiles from natural fibres, which could replace commercial face masks. Their FE was enhanced with the use of electrospun polyvinylidene fluoride (PVDF) polymer fibres. Size- and time-dependent measurements were performed to determine the optimal grammage of the electrospun polymer for effective filtration and the minimum loading of the polymer material.

## 2. Materials and Methods

### 2.1. Filtration Efficiency

All of the FE measurements were performed in a PMMA cubical test chamber, with an edge of 0.6 m. An off-the-shelf medical nebulizer was used to generate the NaCl aerosols in the measured range. A flow of 3.1 L·min^−1^ was used in combination with an aqueous NaCl solution with 2% mass concentration to obtain the desired aerosol particle density. A steady-state aerosol concentration with the peak of the particle size distribution between 40 and 50 nm within the test chamber was ensured before each measurement (Figure S1), which satisfied the EN 13274-7:2019 standard.

The inspired air was sampled with a nanoparticle detector (Scanning Mobility Particle Sizer (SMPS), model 3080 L85; TSI Incorporated, Saint Paul, MN, USA) equipped with a desiccator, soft X-ray neutralizer, long differential mobility analyser, and a water condensation particle counter (WCPC; model 3785; TSI Incorporated, Saint Paul, MN, USA). In this manner, we measured the aerosol time-dependent size distribution, with a 3 min time resolution. The sheath flow rate through the differential mobility analyzer (DMA) was 3.1 L·min^−1^, while the aerosol flow rate through the WCPC was 1 L·min^−1^. The SMPS was operated with the Aerosol Instrument Manager version 9.0.0.0, where the multiple charge correction and the diffusion correction were enabled. The inverted SMPS measurements were further analysed using our own software written in Mathematica. The electrical mobility diameter of counted nanoparticles was from 25.9 to 685.4 nm, which covers the size range of the SARS-CoV-2 virus, with a diameter of around 50–200 nm in the uncovered state, and small respiratory droplets [36]. Each measurement lasted 15 min. The overall FE was determined from the third measurement from the onset of the measurement. Further details on the definitions and calculation specifics can be found in our previous publication [11,12]. The FE was determined using a special holder carrying a 4 cm × 4 cm piece of the material at the wider part and connected with a tube to the SMPS. This design prevented any leakages and ensured that the surface area exposed to the particulate matter was always the same.

### 2.2. Selection of Materials

Five natural materials commonly used in home-made masks were analysed. Their properties are described in Table 1. Each measured sample consisted of two layers of the same material, first without and second with the electrospun fibres sandwiched between the layers. The wefts and warps per length were estimated from SEM images, and number of fibres in the wefts and warps were counted by hand. For the muslin, the numbers in the Table 1 are from one layer, but the fabric is composed of two layers.

### 2.3. Scanning Electron Microscopy

Scanning electron microscopy (SEM) images were made with Prisma E scanning (Thermo Fisher Scientific Inc., Waltham, MA, USA) and JSM 7600F (Jeol Ltd., Tokyo, Japan) microscopes. Each sample was coated with thin layer of Au/Pd to prevent charging effects during electron irradiation. The SEM was used to analyse the size and structure of the fibres of cotton masks.

### 2.4. Polymers and Electrospinning

An 18 wt.% PVDF copolymer (Poly(vinylidene fluoride-co-hexafluoropropylene), Sigma-Aldrich, Merck d.o.o., Slovenia, Europe) in DMF (n,n-dimethylformamide, Carlo Erba Reagents GmbH, Emmendinge, Germany) solution was mixed for three hours at 60 °C prior to electrospinning. The PVDF solution was placed in a plastic syringe fitted with a needle with the orifice 0.8 mm in diameter. A syringe pump (Razel R99-E, Razel Scientific Instruments, St. Albans, VT, USA) was used to feed the solution into the needle at a flow rate of 0.17 mL·h^−1^. The positive output lead of a high-voltage power supply (HVG-P60-R-EU, Linari Engineering, Pisa, Italy) was attached to the needle. An electrically grounded aluminium foil was used as the collector. The applied voltage was 15 kV and the collector-to-needle distance was 20 cm. The mask materials were placed on top of the aluminium foil.

### 2.5. Grammage of Electrospun Fibres

The PVDF polymer was electrospun on a circular substrate with a diameter of 47 mm, which was weighed before and after the electrospinning process. The grammage for each polymer deposition was calculated as the mass difference divided by the area of the substrate. The electrospun PVDF was applied to the samples by placing the sample in the electrospinning device for a certain amount of time. The grammage of the electrospun PVDF as a function of time is shown in Figure S2.

### 2.6. Raman Spectroscopy

The samples were examined with a scanning confocal Raman microscope WITec Alpha 300 RS (WITec GmbH, Ulm, Germany) in backscattered geometry with a polarized Nd:YAG laser operating at the wavelength of 532 nm. The laser beam was focused through a 100 × 0.9 microscope objective. The power of the laser at the sample was approximately 6 mW.

### 2.7. Permeability Measurements

The permeability measurements were performed by Air Permeability Tester FX 3340 MinAir (TEXTEST AG, Schwerzenbach, Switzerland) using EN 14683 standard. The sample area under the test was 4.9 cm^2^ with the air flow reaching 8 L·min^−1^, which is equal to 163.27 L·min^−1^·dm^−2^.

## 3. Results

### 3.1. Time- and Size-Dependent Filtration Efficiency of Different Natural Materials

The overall FE for selected natural materials is shown in Table 2. The S4 sample had the highest overall FE of 88.5%, followed by S2 (43.3%), S1 (38.8%), S5 (25.5%), and S3 (14.5%).

**Figure 1:**
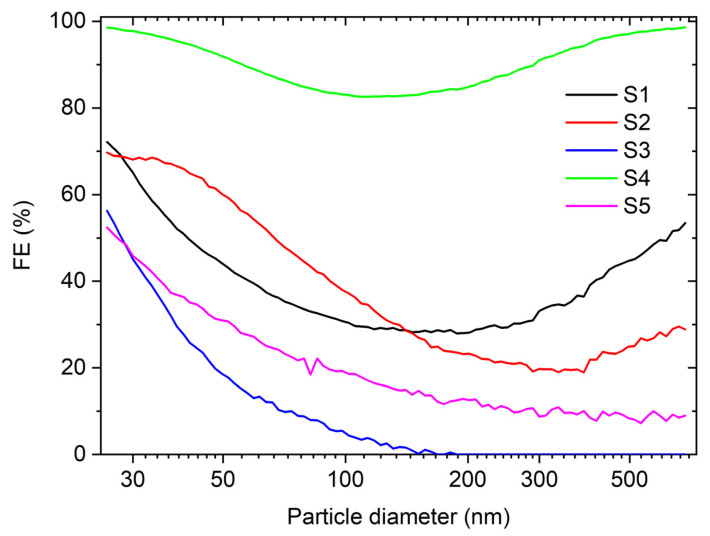
Filtration efficiency as a function of particle diameter for all samples without the electrospun PVDF.

**Figure 2:**
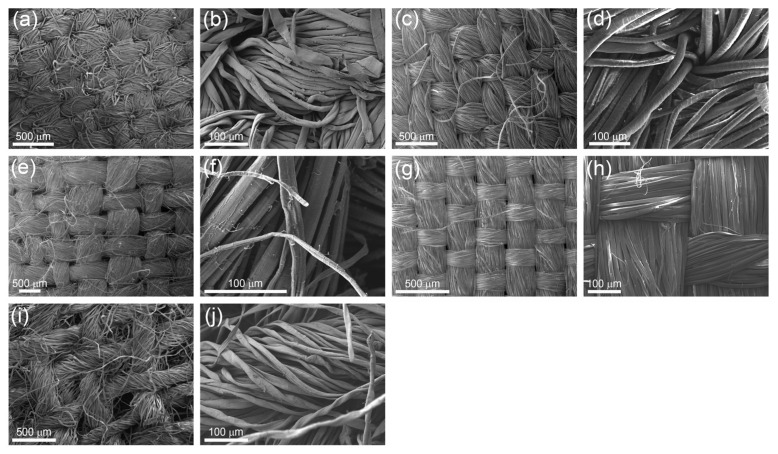
SEM images of sample S1 (**a**,**b**), S2 (**c**,**d**), S3 (**e**,**f**), S4 (**g**,**h**), and S5 (**i**,**j**).

The size-dependent FE for all tested materials is shown in Figure 1. As expected, the highest FE for all particle diameters was for the S4 sample, which is most probably due to the tight mesh of the fabric—Figure 2. Despite its high FE, this material (silk) is the least suitable for masks, as it is almost impermeable to air during breathing. The size-dependent FE of samples S1 and S2 starts at approx. 70% for the particles with a diameter of 25.9 nm and reaches a minimum FE value of 28% at 187.7 nm and 19% at 385.4 nm, respectively. The minimum FE value is in the particle diameter range, where the aerosols are too small for direct impaction, interception, or settling, and too big for diffusion and electrostatic attraction to be an efficient removal mechanism [37]. Similar size-dependent FE measurements were also observed in other studies, where materials from different cloth masks, surgical masks, and facepiece respirators were tested [11]. The low overall FE is mainly due to the large pore size and lack of a static electric charge on the surface of the fibres [12]. The static charge of the electret fibres plays an important role especially for sub-micrometre aerosols, as it retards the progress of the particles, increasing the probability that they impact a fibre due to diffusion and/or the direct impact to the surface of the fibre. For the S3 and S5 samples, the FE starts at approx. 55% for the particles with a diameter of 25.9 nm and continuously drops to 0% and 8%, respectively. The lower FE of the S3 and S5 samples, in comparison to S1 and S2, is attributed to larger pore sizes in the fabric—Figure 2. The studied materials differ in the type of mesh of the fabric as well as in the shape and diameter of the threads and the density of the weaving. The S2 sample threads are between 10 μm and 30 μm in diameter and of a rounded cross-section while all other materials have threads with a similar diameter but with a more flattened cross-section.

To enhance the FE performance, the electrospun PVDF polymer with a grammage of 0.80 mg/m^2^ was sandwiched between two layers of the natural fabrics. The electrospun fibres crystallized mostly in the β phase due to the strong intensity of the 881 cm^−1^ and 839 cm^−1^ peaks [38] present in the Raman spectrum—Figure S3. The β phase possesses the highest dipolar moment per unit cell and shows stable remnant polarization due to stability of the nuclei, thus making the β phase an excellent electret [39].

**Figure 3:**
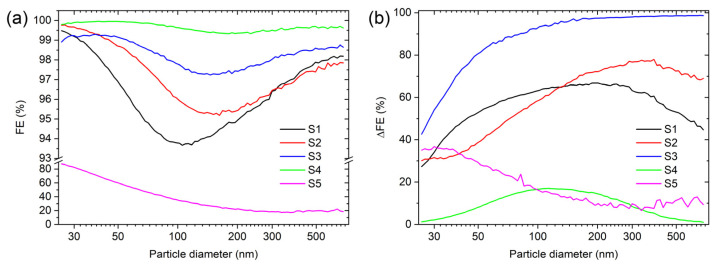
(**a**) FE as a function of particle diameter with the electrospun PVDF. (**b**) The difference in FE (ΔFE) with and without the electrospun PVDF.

The overall FE for the enhanced natural materials is shown in Table 2. The highest overall FE for S4 was 99.7%, followed by S3 (98.2%), S2 (97.3%), S1 (95.8%), and S5 (47.6%). The size-dependent FE and the FE enhancement for all tested materials is shown in Figure 3a. All samples, except S5, have similar line shapes of the size-dependent FE, with the minimum FE situated between 100 nm and 300 nm. The minimum is situated near the most penetrating particle size (MPPS) where all the filtration mechanisms are comparatively inefficient. This agrees with other measurements from the literature as, in this particle diameter range, there are no efficient removal mechanisms [11,12,37]. The S5 sample has also shown increased FE for all particle diameters but the enhancement is smaller compared to other materials. This is better illustrated in the difference of the FE (ΔFE) plot shown in Figure 3b, where the FE of the natural fibres is subtracted from the FE with the electrospun PVDF for each particle diameter. The smallest increase in the FE was for S4, as the sample already had a high FE, even without the electrospun PVDF, while the highest increase in the overall FE was more than 80% for sample S3—Table 2.

**Figure 4:**
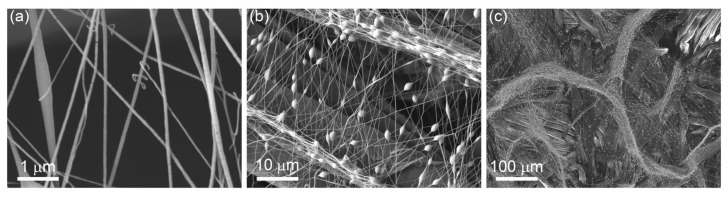
(**a**) Electrospun fibres at high magnification. (**b**,**c**) Lower magnifications of the electrospun fibres on natural materials.

SEM images of the electrospun PVDF fibres on the natural materials are shown in Figure 4. The PVDF fibres are around 110 ± 85 nm in diameter (Figure S4) and are well-distributed over the whole area of the sample—Figure 3.

**Figure 5:**
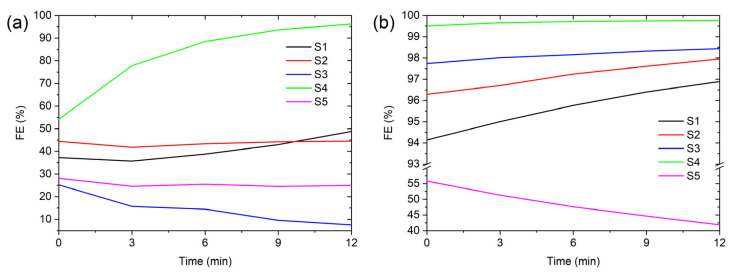
Time-dependent overall filtration efficiency without (**a**) and with (**b**) electrospun PVDF fibres.

Time-dependent FE measurements for the samples with and without the electrospun PVDF are shown in Figure 5. For the S4 and S1 samples without the electrospun PVDF, the total FE increases with time; for S3, it decreases; while for S2 and S5, the FE does not change with time. For the samples with the electrospun PVDF, the total FE increases with time except for S5, which decreases—Figure 5b. The reason for the increase in the total FE over time could be the accumulation of NaCl crystals on the surface of the electrospun PVDF, which act as an additional barrier for other aerosols [11]. The accumulation of NaCl particles can be seen in Figure S5. Particles smaller than the holes between individual fibres are captured as well. These particles are attracted to the fibres due to the electric and/or dielectrophoretic forces or hit the fibre due to inertia. Once in contact with the fibre, the van der Waals force keeps them in place. In the samples, where the FE did not increase with time, the aerosol particles went through the filtering material unobstructed. This is probably due to the larger pore size in the natural material. Larger pores would allow the airflow, which carries the aerosols, to follow a less obstructed path, which in turn would decrease the probability for an aerosol to come in contact with the filter surface.

**Figure 6:**
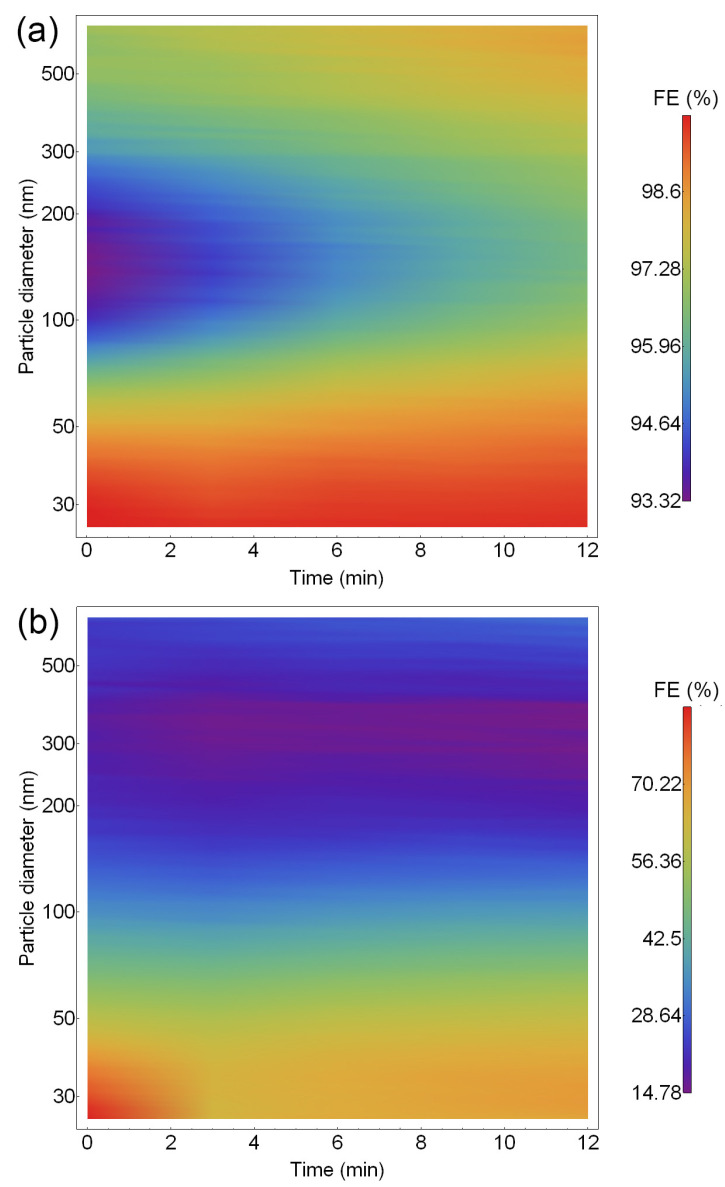
Filtration efficiency vs. particle diameter in log scale vs. time for (**a**) with PVDF fibres and (**b**) without the fibres.

The increase of the FE with time is due to the increased FE for particles near the MPPS—Figure 6a. In the case of sample S2 without the electrospun polymer, the filtration efficiency for the individual particle diameter does not change with time—Figure 6b. With the addition of the eletrospun polymer, the FE increases for all particle diameters but is also time-dependent. For particle diameters between 100 nm and 300 nm, the FE increases for several within the 15 min measurement. Similar results are seen in all other samples, where the FE increased with time.

### 3.2. Time- and Size-Dependent FE Measurements for Different Electrospun Poylmer Thicknesses

To test if the grammage of the electrospun PVDF influences the FE, different grammages were applied to the same natural material. In our case, we used cotton (sample S1), as it is the most widely used material for home-made masks. The overall FE for four grammages are presented in Table 3. The overall FE increases with the grammage of the electrospun PVDF. With the grammage of 0.19 mg/m^2^, the overall FE increased from 38.8% to 82.6%. The 43.8% increase in FE is attributed to the finer mesh of the PVDF fibres compared to the natural fibres, and their surface charge. A similar increase in the overall FE was observed also in other studies where electrospun PVDF fibres were used in combination with cotton [22]. By further increasing the grammage to 0.80 mg/m^2^, the overall FE increased to 95.8%, which is comparable to the overall FE of surgical masks [11]. With the grammage of the electrospun PVDF of 2.69 mg/m^2^, the overall FE is 99.9%, which is comparable to the overall FE for the fabrics of FFP2 and FFP3 facepiece respirators [11]. By increasing the grammage, the PVDF fibres form a thicker layer, increasing the path the aerosols travel though, which increases the chance that the aerosol will be captured.

**Figure 7:**
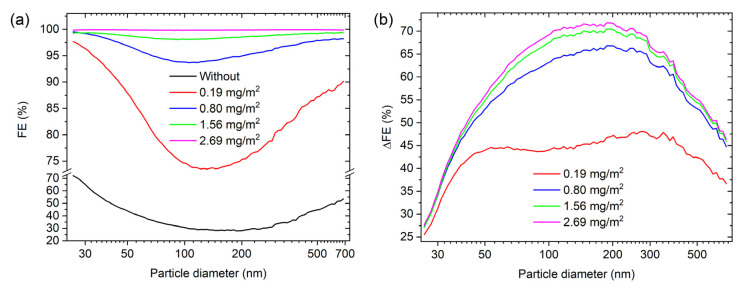
(**a**) Filtration efficiency as a function of the particle diameter; (**b**) ΔFE as a function of the particle diameter.

As seen from the size-dependent measurements shown in Figure 7, the FE for each particle diameter increases with the increasing grammage of the electrospun polymer. To better visualize the change in the FE for each thickness, a ΔFE plot is shown in Figure 7. The biggest increase in the FE is seen for the grammage of 0.19 mg/m^2^. The FE is further increased for the grammage of 0.80 mg/m^2^, while for the grammage of 1.56 and 2.69 mg/m^2^, the ΔFE are similar. The particle diameter at which the minimum of the FE is reached decreases with thickness, from 190 nm for the sample without the electrospun PVDF to 90 nm for the grammage of 2.69 mg/m^2^. The time-dependent measurements show that the overall FE increases with time—Figure S6. The result is expected as a similar trend was seen with most of the samples with the electrospun PVDF.

Although the FE increases with the amount of electrospun PVDF, the pressure difference also plays an important role when evaluating the performance of filtration materials. The measured pressure differences for the samples with different grammages are presented in Table 3. In our case, the pressure difference was lowest for the sample with the grammage of 0.80 mg/m^2^ and highest for the sample with the grammage of 2.69 mg/m^2^ where both measurements deviated for approx. 20% compared to the sample without electrospun fibres. This indicates that the addition of electrospun fibres in the applied grammage range does not substantially affect the pressure difference of the filtration material. By using a more permeable natural material, the pressure drop could be reduced while the FE could still be enhanced. The small changes in the pressure difference and large increase in the FE is also reflected in the quality factor of the samples, presented in Table 3. The quality factor increases with increasing grammage mostly due to the higher FE.

By optimising the grammage of the electrospun polymer, the FE of the material can be precisely controlled for a specific application—Figure S7. By optimising the amount of the used polymer, the environmental impact could be minimized.

## 4. Conclusions

Wearing a face mask is considered one of the most effective ways to mitigate the spread of airborne pathogens. The mass use of such masks creates large quantities of waste which could pollute the environment if not disposed of properly. One of the possible solutions to make them more environmentally friendly are masks made of natural materials. However, natural materials do not possess an intrinsic surface charge, resulting in low filtration efficiency (FE) and rendering them unusable. By applying a thin layer of electret, such as PVDF fibres, to natural materials, their overall FE is greatly increased. In this study, we measured the FE of five different natural materials in combination with electrospun PVDF fibres. The FE substantially increased when the PVDF fibres were added. The increase in the FE is attributed to the finer mesh and surface charge of the electrospun fibres. The electret fibres greatly increase the removal of MPPS particles with diameters between 100 and 300 nm. By optimising the grammature of the electrospun fibres, the overall FE can be tuned to a specific application while adding only a minimal amount of the polymer. Pressure drop measurements show small changes within the applied grammage range indicating that with the right selection of the material, the pressure drop could be minimized while the FE could be enhanced, increasing the overall quality factor. However, the selected cotton fabric has a relatively high pressure drop. Thus, the cotton–PVDF filtration material does not comply with the EN 14683 standard for face masks due to the high pressure drop values. Although proof-of-concept has been demonstrated, further improvements in the design and natural material selection is needed before the composite can be used for face masks. By using natural materials in combination with a relatively small amount of the polymer, the environmental impact of such masks would be smaller compared to standard masks and respirators made only of polymers.

## Figures and Tables

**Table 1:** Sample name, material, and its grammage. For each sample, the same grammage of the electrospun fibres was used.

Sample Name	S1	S2	S3	S4	S5
Material	Cotton	Wool	Linen	Silk	Muslin
Grammage (g/m^2^)	140	150	190	90	130
Nbr. of fibres in weft/warp	90/80	50/40	280/220	140/40	60/60
Wefts/Warps per length (mm^−1^)	2.4/4.1	3.4/3.4	1.4/1.7	3.6/4.8	2.1/2.1
Grammage of electrospun PVDF (mg/m^2^)	0.19, 0.46, 0.80, 1.56, 2.69	0.80	0.80	0.80	0.80

**Table 2:** The overall FE without and with the electrospun PVDF fibres and the overall FE difference (ΔFE).

Sample	S1	S2	S3	S4	S5
FE (%)—without	38.8	43.3	19.5	88.5	25.5
FE (%)—with	95.8	97.3	98.2	99.7	47.6
ΔFE (%)	57.0	54.0	83.7	11.2	22.1

**Table 3:** The grammage of the electrospun PVDF on cotton with their corresponding overall FE, pressure difference, and quality factor calculated according to [39].

Grammage (mg/m^2^)	Without	0.19	0.80	1.56	2.69
FE (%)	38.8	82.6	95.8	98.6	99.9
Pressure difference (Pa)	500	455	407	485	613
Quality factor (kPa^−1^)	1	3.8	7.8	8.8	11.3

## Data Availability

Data are available upon request from the authors.

## Supplementary Materials

The following supporting information can be downloaded at: https://www.mdpi.com/article/10.3390/ma16062314/s1. Figure S1: Normalized number concentration of the steady state conditions before the measurement. Figure S2: The grammage as a s function of the electrospinning time. Figure S3: Raman spectrum of the electrospun PVDF polymer. Figure S4: A histogram of the electrospun fibre diameter. Figure S5: NaCl particles adhered to PVDF nanofibers after the filtration efficiency measurement. Figure S6: The time dependence of the filtration efficiency for different grammages of electrospun PVDF on cotton fabrics. Figure S7: Filtration efficiency as a function of grammage.
