# Supplementary material for: Enhanced Filtration Efficiency of Natural Materials with the Addition of Electrospun Poly(vinylidene fluoride-co-hexafluoropropylene) Fibres
Source: Materials (Basel). 2023 Mar 14;16(6):2314. doi: 10.3390/ma16062314 (PMC10054789; doi:10.3390/ma16062314)
Supplement: Supplementary file 1 [file materials-16-02314-s001.zip › materials-2194618-supplementary.pdf]

Enhanced Filtration Efficiency of Natural Materials with the Addition of Electrospun Poly(vinylidene fluoride-co-hexafluoropropylene) Fibres

Gregor Filipič [1], Luka Pirker [1,2,*], Anja Pogačnik Krajnc [1,3], Marjan Ješelnik [4] and Maja Remškar [1]

1 Jozef Stefan Institute, Jamova cesta 39, 1000 Ljubljana, Slovenia; gregor.filipic@ijs.si (G.F.)
2 Department of Electrochemical Materials, J. Heyrovsky Institute of Physical Chemistry, Dolejškova 3, 182 23 Prague, Czech Republic
3 Faculty of Mathematics and Physics, University of Ljubljana, Jadranska ulica 19, 1000 Ljubljana, Slovenia
4 smartMelamine d.o.o., Tomšičeva cesta 9, 1330 Kočevje, Slovenia
* Correspondence: luka.pirker@ijs.si

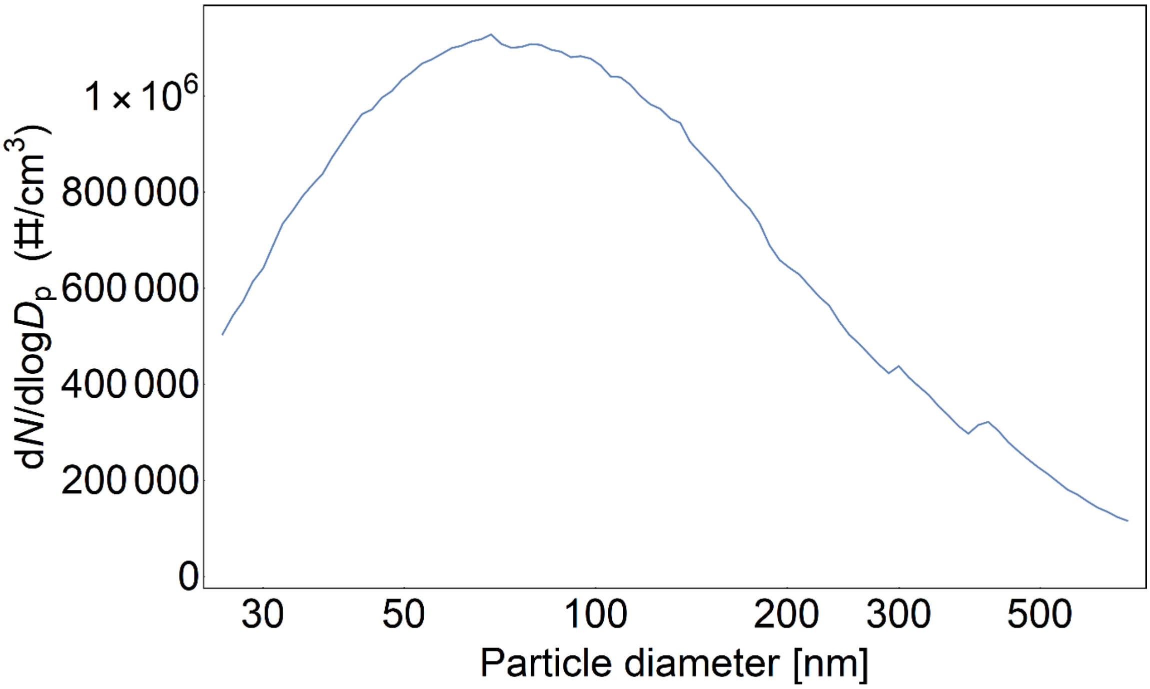

Figure S1. Normalized number concentration of the steady state conditions before the measurement.

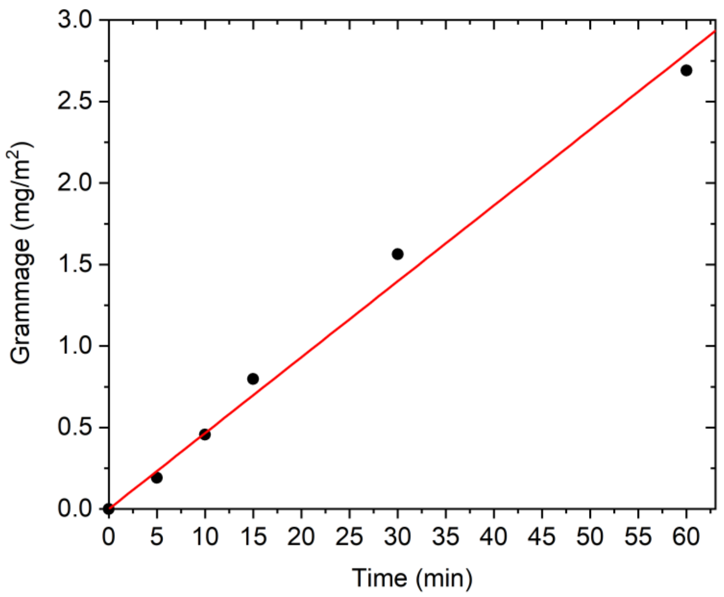

Figure S2. The grammage as a s function of the electrospinning time.

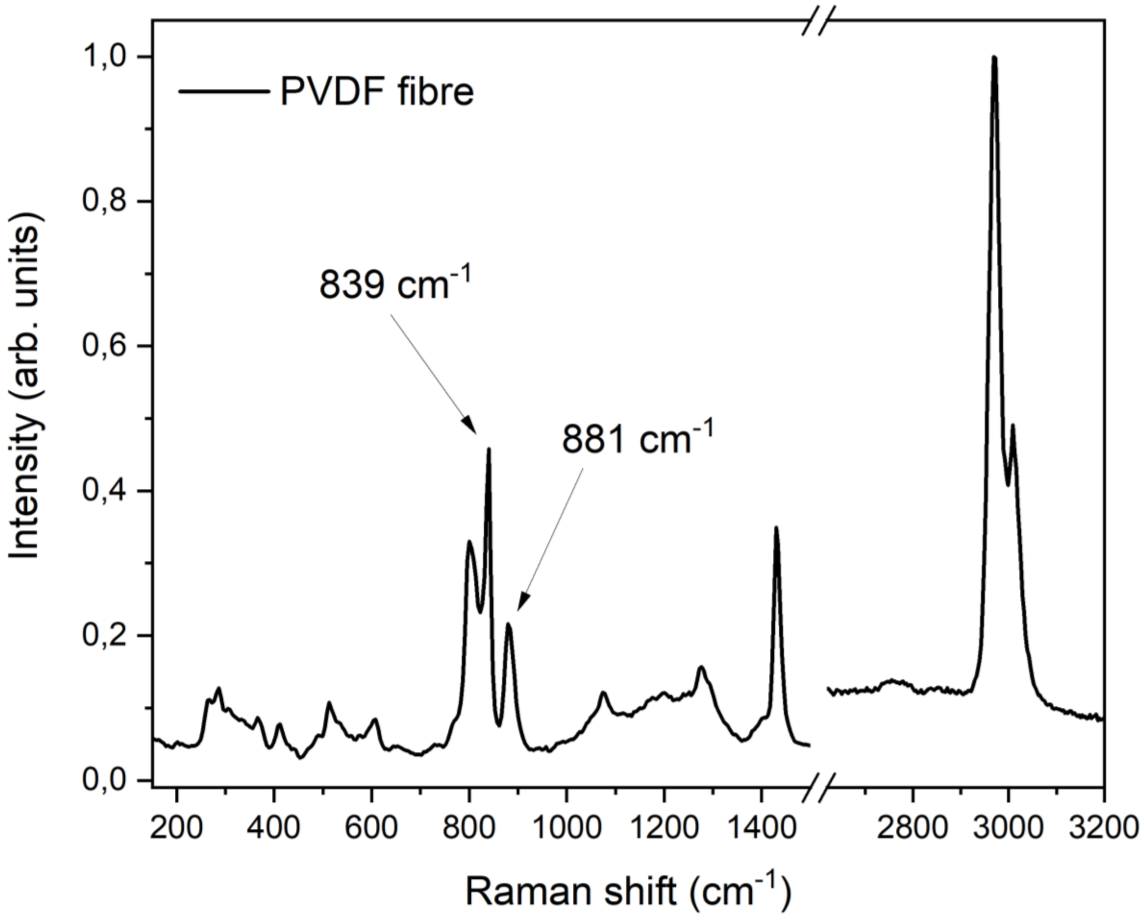

Figure S3. Raman spectrum of the electrospun PVDF polymer.

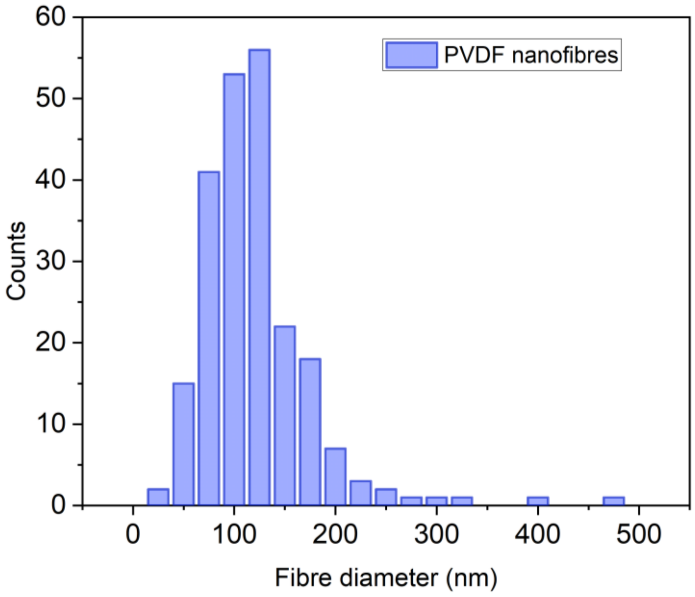

Figure S4. A histogram of the electrospun fibre diameter.

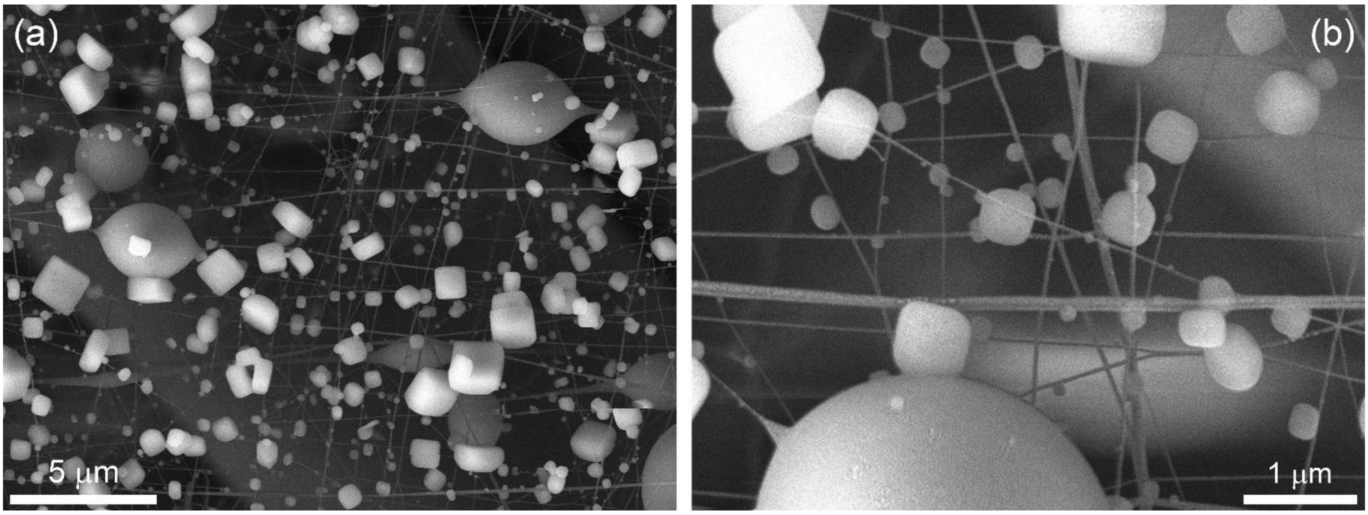

Figure S5. NaCl particles adhered to PVDF nanofibers after the filtration efficiency measurement.

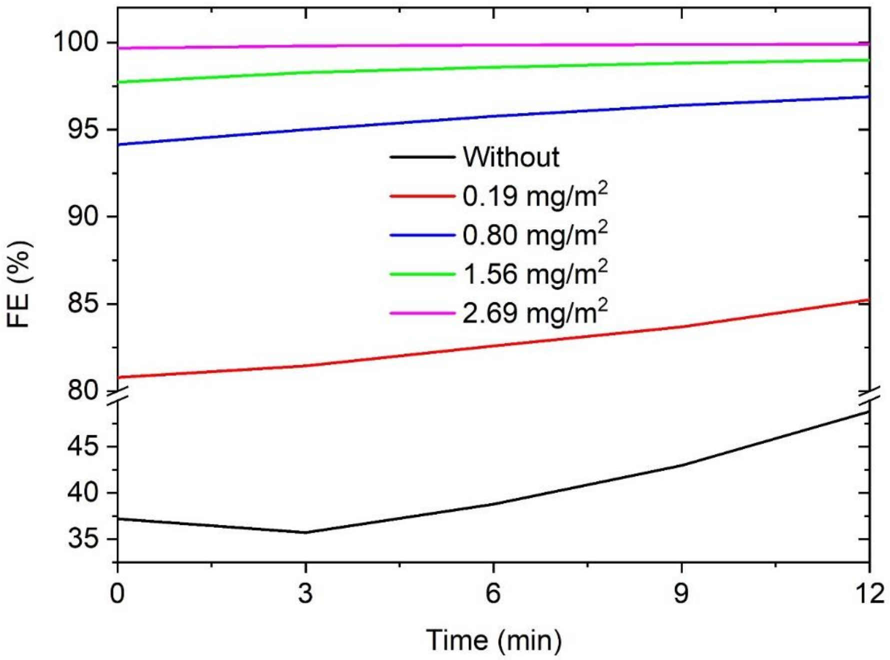

Figure S6. The time dependence of the filtration efficiency for different grammages of electrospun PVDF on cotton fabrics.

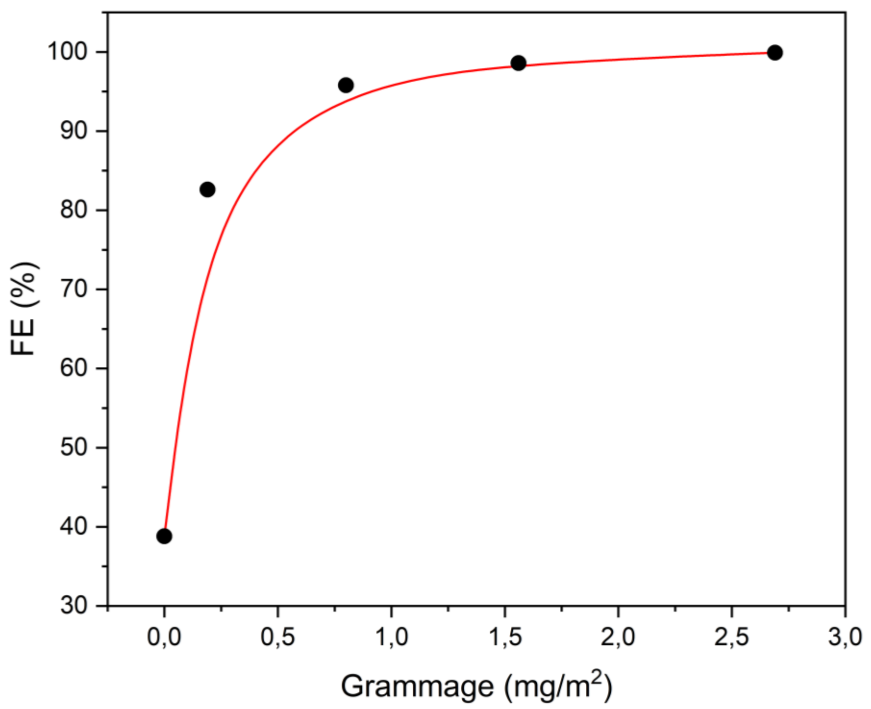

Figure S7. Filtration efficiency as a function of grammage.
